# Highlight: The Evolutionary Arsenal of Aphids

**DOI:** 10.1093/gbe/evy217

**Published:** 2018-10-13

**Authors:** Casey McGrath

Nature provides countless examples of evolutionary arms races, in which species develop adaptations and counter-adaptations in a struggle for survival and reproduction. Such arms races are common between predator and prey or between parasite and host. Understanding this coevolutionary process can aid in our ability to develop necessary countermeasures, such as overcoming bacterial resistance to antibiotics.

Aphids are a diverse group of herbivorous insects. Over 5,000 aphid species have been described, and over 500 are serious agricultural pests, threatening global food production. Aphids feed by pushing their needle-like mouthparts into a plant and sucking the sap, or phloem. In addition to taking nutrients directly from the plant, effector proteins in aphid saliva are transmitted to the plant and act to combat the plant’s defense responses. This establishes the basis for an evolutionary arms race between aphids and host plants, with plants evolving systems to detect and disrupt aphid effectors, and aphids evolving new effectors capable of evading plant defense mechanisms.

In order to better understand the success of these pests, researchers have sought to identify aphid effector proteins and shed light on how they evolve. Thanks to advances in genome and transcriptome sequencing, as well as bioinformatics and proteomics, an unprecedented number of aphid effectors are now being identified. In a new report in *Genome Biology and Evolution*, “Shared transcriptional control and disparate gain and loss of aphid parasitism genes” (Thorpe et al. 2018), Sebastian Eves-van den Akker of the University of Cambridge and Jorunn Bos of the University of Dundee, along with their colleagues, reveal new insights into the mechanisms of aphid effector evolution and regulation.

In their study, Thorpe et al. sequenced the genomes of two aphid species: *Myzus cerasi*, which parasitizes stone fruit plants like the cherry, and *Rhopalosiphum padi*, which feeds on cereals. They then used data from these two species along with three previously sequenced aphid genomes to determine patterns of gene and genome evolution. “Excitingly, we found that a combination of distant gene duplication, recent gene birth, and disparate gain and loss of genes have contributed to aphid genome evolution in general, and effector repertoires in particular,” says Bos.

After identifying candidate effector genes, the authors noticed something unusual: two specific effector genes were tightly physically linked in all five of the aphid genomes studied and were also highly coregulated in *R. padi*, suggesting that they are linked functionally. Thorpe et al. then looked for other genes that shared the same expression patterns and identified hundreds of other genes distributed across the genome that were coregulated with the original pair of genes. For Eves-van den Akker, this exciting finding indicates “the discovery of a shared transcriptional control system of putative aphid effectors.” He believes that there is a common genetic or epigenetic element that links these genes and facilitates their coexpression. Moreover, the authors point out that this discovery should help researchers identify aphid effectors in the future, which can be tedious and time-consuming, according to Bos. “By improving selection criteria, we can develop a better way to prioritize predicted effectors for functional assays.”

In addition to this coordinated regulation, the rapid evolution of effectors may be beneficial in arming aphids against plant defense systems. Indeed, in another recent paper in *Genome Biology and Evolution* (Boulain et al. 2018), an independent group of researchers including Jean-Christophe Simon and Akiko Sugio found that candidate effector genes tended to evolve faster than the average aphid gene. As with many new models, however, some controversy exists: Simon and Sugio have reservations about the transcriptional control system of effectors proposed by Thorpe et al., noting that “because of the experimental design, putative effectors are already selected by being upregulated in the head vs. the body.” Nevertheless, they believe that these two studies together show that the aphid “effector repertoire has been very likely shaped along the evolutionary course of antagonistic relationships between aphids and plants.”

Thus, current research points to dynamic gene birth/loss, rapid evolution, and potential coregulation as weapons in the aphid’s evolutionary arsenal. Importantly, such research lays a foundation for the development of methods to control these agricultural pests. According to Bos, “We are keen to find out what the mechanism is that controls transcription of a large set of aphid effectors. This…would allow us to explore how to disrupt regulation as a way to control aphid infestations.” In addition, Bos notes that future research should focus on discovering the detailed functions of different effector proteins from different species. While this is likely to be time-consuming, Bos believes “this represents an important research area where progress is needed to understand how plants and aphids interact at the molecular level.”
